# The Factors Associated With Telehealth Use and Avoidance During the COVID-19 Pandemic: Longitudinal Survey

**DOI:** 10.2196/43798

**Published:** 2023-02-08

**Authors:** Louise M Farrer, Philip J Batterham, Amelia Gulliver, Alyssa Morse, Alison L Calear, Sonia McCallum, Michelle Banfield, Yiyun Shou, Eryn Newman, Amy Dawel

**Affiliations:** 1 Centre for Mental Health Research The Australian National University Canberra Australia; 2 Research School of Psychology The Australian National University Canberra Australia; 3 Saw See Hock School of Public Health National University of Singapore Singapore Singapore; 4 Lloyd's Register Foundation Institute for the Public Understanding of Risk National University of Singapore Singapore Singapore

**Keywords:** telehealth, access, inequity, health care, COVID-19, telemedicine, health care service, longitudinal survey, health care system, technology acceptance, health care delivery, Australian population

## Abstract

**Background:**

Social distancing requirements due to the COVID-19 pandemic saw a rapid increase in the delivery of telehealth consultations as an alternative to face-to-face health care services.

**Objective:**

The aims of this study were to assess the use and acceptability of telehealth during the early stages of the pandemic and identify factors associated with telehealth avoidance during this period.

**Methods:**

Data were obtained from waves 4 and 7 of a longitudinal survey designed to assess the impact of the COVID-19 pandemic on the health and behavior of a representative sample of Australian adults. Participants reported on their use or avoidance of telehealth during the assessment period, as well as the mode of telehealth used and acceptability.

**Results:**

Approximately 30% of participants reported using telehealth during the assessment periods, with the most common telehealth modality being the telephone. Acceptance of telehealth was generally high and was higher among those who used telehealth compared with those who did not. Approximately 18% of participants reported avoiding health care due to telehealth. Across assessment waves, avoidance was associated with younger age, speaking a language other than or in addition to English, having a current medical diagnosis, and lower levels of telehealth acceptability.

**Conclusions:**

While most participants in this study were accepting of telehealth services, there remain barriers to use, especially among those from particular sociodemographic groups. At a population level, avoidance of health services in nearly one in five adults may have considerable long-term impacts on morbidity and potentially mortality. Targeted efforts to promote engagement with telehealth services are critical if these adverse outcomes are to be avoided, particularly during periods when access to face-to-face services may be limited.

## Introduction

There has been a substantial increase in the uptake of telehealth since the onset of the COVID-19 pandemic [1]. During the early stages of the pandemic in 2020, social distancing policies, limitations placed on surgery, and avoidance of face-to-face care, such as for screening procedures, combined to substantially reduce the frequency of in-person health care [2]. Concurrently, health care services and medical practitioners adapted rapidly to telehealth to enable the continuation of care while also protecting staff and patients from COVID-19 infection [3]. While face-to-face delivery is necessary for certain procedures such as physical examinations, medical tests, and surgery [4], telehealth is a viable alternative for many health care services. Continuity of care during the pandemic was particularly important for people with illnesses and chronic conditions requiring regular monitoring and treatment [5-7].

A substantial evidence base for the effectiveness of telehealth existed prior to the pandemic [8]. For specific medical fields such as mental health, decades of research have shown the equivalence of telehealth compared with face-to-face treatment, particularly for the most common mental disorders of depression and anxiety [9]. However, the acceleration of telehealth uptake across multiple medical fields during the pandemic has provided an opportunity for significant innovation, particularly in areas that normally would not deliver telehealth, such as emergency departments [10]. Barriers to the use of telehealth among service providers have historically included difficulties using technology among staff, resistance to change, and the financial costs of use. Among health care users, age, educational level, and access to technology have been commonly reported as barriers [11,12]. In Australia, rural and remote communities substantially benefit from the adoption of telehealth; however, issues with how these services are funded has previously caused barriers to uptake [13,14]. For example, many telehealth services provided by allied health in these regions were found in a systematic review not to be eligible for reimbursement through government universal health schemes [14]. However, the rapid changes in service delivery and government funding models [15] during the pandemic may have alleviated some of these barriers, marking a potentially substantial shift toward greater telehealth funding and adoption.

For those who use telehealth services, satisfaction levels have generally been high, both before and during the pandemic [16]. In Australia and internationally, service users commonly report that telehealth appointments are convenient and create savings in travel, time, and money [16,17]. Positive experiences are facilitated by clinicians making an effort to be engaging and establish rapport, the presence of an existing relationship between the patient and clinician, and appropriate appointment types that suit the distal format (eg, routine consultations, managing pre-existing conditions, or updating prescriptions) [16,18]. Since the onset of the COVID-19 pandemic, telehealth services have been offered to a wider range of people in Australia than would normally access them, including through the replacement of their usual face-to-face services. Satisfaction with telehealth service experiences has generally remained high, and distal health care has been perceived as a safer option, allowing continued access to care while preventing potential COVID-19 infection [19-23]. However, willingness to continue to use telehealth services outside of the pandemic context is mixed [21-23].

Telehealth may particularly appeal to people who benefit most from its advantages, for example, those living greater distances from hospitals and specialist services [19,23,24], people with caring responsibilities [17], and people with health conditions or disabilities that make travel or face-to-face appointments more difficult [17,22]. Gender may also interact with some of the aforementioned factors; for example, women are more likely to perform caring roles [25]. In Australia, the perceived disadvantages of telehealth in comparison to face-to-face services (eg, poorer communication, difficulties accessing prescriptions, and pathology results) [20,21,23] may outweigh the benefits for people living in urban areas, closer to service locations, and with easy access to travel [20,23]. Telehealth can also be seen as inappropriate or less effective for certain kinds of appointments or concerns, for example, where physical examination may be necessary or beneficial, or when a new diagnosis is being made [18,21]. In these circumstances, patients may experience anxiety about important health information being missed in a phone call or video consultation [19].

Further, the common barriers to accessing telehealth services, noted above, may prevent people from using telehealth services at all. Research from the United States suggests that during the pandemic certain disadvantaged groups were less likely to access telehealth services, including people without health insurance, people living in a mobile home or trailer, and people living in areas with poorer broadband internet coverage [26]. This is particularly problematic as disadvantaged groups may stand to benefit most from many of the financial and logistical benefits that telehealth can provide [23]. A recent Australian study did not find an association between socioeconomic status and preferences for telehealth services. However, the participants of this study were people who had recently accessed these services, and this study did not use a representative sample [23]. Another survey found that Australians who accessed telehealth services were more likely to be older, be female, have attained a higher level of education, have a greater prevalence of chronic conditions, and have poorer general health compared to those who did not access them [21]. However, these results need replicating in a representative sample.

Much of the research on attitudes toward the use of telehealth focuses on the experiences of people who have used such services. Thus, it is unclear who may have avoided or been unable to access telehealth services during the pandemic and what drove their choice or ability to do so. Therefore, this study aimed to investigate the use and acceptability of telehealth in a representative sample of adults during the early stages of the COVID-19 pandemic. Specifically, we sought to investigate the proportion of participants who had used telehealth and the type of modality used, views on telehealth acceptability and reasons for future use of telehealth, the proportion of people who avoided health care because of having to use telehealth during the pandemic, and factors that were associated with avoidance of telehealth. These findings may improve our understanding of factors driving the use and avoidance of telehealth, and inform strategies to improve telehealth acceptability and uptake.

## Methods

### Ethics Approval

The ethical aspects of the study were approved by The Australian National University Human Research Ethics Committee (protocol 2020/152), and the full study protocol is available online [27].

### Participants and Procedure

Data for the study were obtained from a longitudinal survey designed to assess the impact of the COVID-19 pandemic on the health and behavior of a representative sample of Australian adults (The Australian National COVID-19 Mental Health, Behaviour and Risk Communication Survey) [28]. The survey was administered to research panels managed by Qualtrics Research Services over 8 waves, with the first 7 waves completed on a fortnightly basis between March and June 2020, and the eighth wave completed in March 2021. Participants were emailed an invitation to complete each survey wave and were provided a 1-week window to complete it, with up to 5 email reminders sent during this period. Quota sampling was used to obtain a sample of the Australian population that was representative based on age group, gender, and state/territory of residence. Online written informed consent was obtained online from all participants prior to participation in the study.

The first wave of data collection commenced on March 28, 2020 (N=1296), and telehealth use data were collected at wave 4 (May 9, 2020) and wave 7 (June 20, 2020). At wave 7, 58.3% (n=756) of the sample was retained. Data for this study were derived from wave 1 (demographics) and waves 4 and 7 (telehealth use and acceptability). However, data were analyzed and reported only for participants with complete data on the telehealth variables of interest at waves 4 and 7 (n=706).

### Measures

#### Telehealth Use and Acceptability

Participants were asked to indicate how many telehealth appointments they had over the last month (wave 4) or 6 weeks (wave 7). Participants were also asked to indicate the modality of their telehealth appointments (videoconferencing, talking/texting using a telephone, online text chat/email) and whether they had avoided or delayed accessing health care as a result of having to use telehealth. Telehealth acceptability was assessed by asking participants to indicate their agreement (on a 5-point scale from 1 “strongly disagree” to 5 “strongly agree”) with the following items: “I am confident in my ability to use telehealth to access healthcare”; “I am not, or would not be, concerned about the security of my personal information during a telehealth appointment”; “I feel like the quality of care is just as high in a telehealth appointment as it is in a face-to-face appointment”; “I would be willing to access healthcare via telehealth in the future”; and “Telehealth is useful in exceptional circumstances like COVID-19, but not at other times.” Those who agreed with the statement that they would be willing to access telehealth in the future were asked for their reasons why (“it’s convenient,” “it works well/is effective,” “to protect my health,” “other”). Participants were also asked to indicate their confidence in using technology (on a 5-point scale ranging from 1 “not at all confident” to 5 “extremely confident”).

#### Demographic Characteristics

The following demographic characteristics were measured at wave 1 and included in this study: age, gender (male/female), education (years), lives alone (yes/no), current diagnosis of a physical or mental health condition (yes/no), a language other than or in addition to English spoken at home (yes/no), and remoteness (major city vs regional/remote/very remote).

### Statistical Analyses

Analyses were conducted using SPSS v.27 (IBM Corp). Demographic characteristics, telehealth use, and acceptability were analyzed and reported descriptively. An exploratory factor analysis was conducted on the 5 items assessing telehealth acceptability with the aim of creating a continuous telehealth acceptability variable for further analysis. Logistic regression was used to examine the following predictors of health care avoidance due to telehealth at waves 4 and 7 (age, gender, remoteness, education, living situation, languages spoken, current medical diagnosis, confidence with technology, and telehealth acceptability). Variables were entered into the models simultaneously.

## Results

### Participants

Table 1 shows the demographic characteristics of participants who completed the telehealth measures at waves 4 and 7 (n=706). The sample in this study was comparable to those who were excluded (n=590) in terms of gender, years of education, and remoteness. However, compared to those excluded, the current sample was older (t_1294_=11.87; *P*<.001), more likely to live alone (*χ*^2^_1_=6.1; *P*=.01), less likely to speak a language other than or in addition to English at home (*χ*^2^_1_=22.2; *P*<.001), and more likely to have a current diagnosis of a health condition (*χ*^2^_1_=13.0; *P*<.001).

Just over half of the participants (n=382, 54.1%) reported a current diagnosis of a physical or mental health condition. Just over two-thirds of participants reported feeling fairly or extremely confident in their use of technology, and the majority had no telehealth appointments during the data collection period. The telephone was the most commonly used telehealth modality. Overall, avoidance of health care due to telehealth was approximately 18% at both waves, and avoidance did not differ between waves (*χ*^2^=0.009; *P*=.93).

**Table 1:** Demographic characteristics and telehealth use of participants (n=706).

			Wave 1		Wave 4	Wave 7
Age (years), mean (SD)			50.99 (15.96)		—^a^	—
Gender (female), n (%)			338 (47.9)		—	—
Education (years), mean (SD)			14.51 (1.77)		—	—
Lives alone (yes), n (%)			100 (14.2)		—	—
Lives in major city, n (%)			548 (77.6)		—	—
Language other than/in addition to English (yes), n (%)			93 (13.2)		—	—
Current diagnosis of a health condition/illness (yes), n (%)			382 (54.1)		—	—
Fairly/extremely confident using technology, n (%)			—		484 (68.6)	—
**Number of telehealth appointments, n (%)**						
	None	—		497 (70.4)		477 (67.6)
	1-2	—		184 (26.1)		186 (26.3)
	≥3	—		25 (3.5)		43 (6.1)
**Telehealth appointment modality, n (%)^b^**						
	Videoconference	—		39 (18.6)		44 (19.2)
	Telephone (talk, text)	—		179 (85.6)		204 (89.0)
	Other online (email, chat)	—		21 (10.0)		23 (10.0)
Avoided health care because of telehealth (yes), n (%)			—		—	124 (17.6)

^a^Not applicable.

^b^Respondents could select more than one telehealth modality. Percentages calculated from number of respondents who had a telehealth appointment (n=209 in wave 4, n=229 in wave 7).

### Telehealth Acceptability

Table 2 shows descriptive analyses of the telehealth acceptability items by use of telehealth. Analysis of the individual acceptability items revealed that the lowest level of agreement at both waves was associated with the statement that telehealth is as high quality as face-to-face care (n=250, 35.4% at wave 4 and n=254, 36% at wave 7). Just over half of the participants at both waves agreed that telehealth has limited utility beyond the pandemic (n=399, 56.5% at wave 4 and n=373, 52.8% at wave 7). Among those who indicated a desire to access telehealth in the future, the most commonly reported reason for this was convenience (n=208, 54.9%). Percentage agreement with the acceptability items was consistently higher among those who had used telehealth compared to those who had not. This was particularly marked for the item measuring confidence in the ability to use telehealth (n=183, 80% vs n=280, 58.7% at wave 7).

With the aim of consolidating the acceptability data for further analysis, acceptability items at waves 4 and 7 were analyzed using principal component analysis with varimax rotation. Loadings of the items on the rotated factor solution are shown in Table 3. A two-factor solution emerged at both waves based on eigenvalues greater than 1 and inspection of the scree plot. Together, the two factors explained 67% of the total variance at wave 4 and 69.2% of the total variance at wave 7. As shown in Table 3, items 1 to 4 loaded satisfactorily on factor 1 (as indicated by factor loadings >0.6), and item 5 loaded on factor 2. A single-factor solution was retained, and the item loading on factor 2 (item 5) was excluded from the scale. Items 1 to 4 were summed to create a total telehealth acceptability score (ranging from 4 to 20, with higher scores indicating higher acceptability). Cronbach α was acceptable for the scale items at each wave (.75 at wave 4 and .78 at wave 7). The mean telehealth acceptability scores did not differ significantly between waves 4 and 7 (t_703_=0.14; *P*=.89). Acceptability was significantly higher among those who had used telehealth compared to those who had not, both at wave 4 (t_703_=4.95; *P*<.001) and wave 7 (t_703_=5.88; *P*<.001).

**Table 2:** Telehealth acceptability.

			Wave 4							Wave 7				
			Used telehealth (n=209)		Did not use telehealth (n=497)		Total (n=706)		Used telehealth (n=229)			Did not use telehealth (n=477)		Total (n=706)
**Acceptability items, n (%)**														
	Confident in ability to use telehealth (agreed)	163 (78.0)		286 (57.6)		449 (63.6)		183 (80.0)			280 (58.7)		463 (65.6)	
	Not concerned about security of telehealth (agreed)	127 (60.8)		259 (52.1)		386 (54.7)		153 (66.8)			234 (49.0)		387 (54.8)	
	Telehealth quality is just as high as face-to-face (agreed)	87 (41.6)		163 (32.8)		250 (35.4)		106 (46.3)			148 (31.0)		254 (36.0)	
	Willing to access telehealth in the future (agreed)	134 (64.1)		255 (51.3)		389 (55.1)		153 (66.8)			226 (47.4)		379 (53.4)	
	Telehealth only useful during COVID-19 (agreed)	125 (59.8)		274 (55.1)		399 (56.5)		124 (54.1)			249 (52.2)		373 (52.8)	
Total acceptability score, mean (SD)			14.48 (3.04)		13.21 (3.09)		13.59 (3.13)		14.59 (3.00)			13.1 (3.23)		13.58 (3.22)
**Why use telehealth in the future? n (%)**														
	Convenience	—^a^		—		—		80 (52.3)			128 (56.6)		208 (54.9)	
	Effective/works well	—		—		—		45 (29.4)			24 (10.6)		69 (18.2)	
	To protect my health	—		—		—		12 (7.8)			18 (8.0)		30 (7.9)	
	Other	—		—		—		7 (4.6)			5 (2.2)		12 (3.2)	

^a^Not applicable.

**Table 3:** Factor loadings for telehealth acceptability items.

Item	Wave 4			Wave 7	
	Factor 1	Factor 2	Factor 1		Factor 2
1. I am confident in my ability to use telehealth to access health care	.78	.18	.74		.07
2. I am not or would not be concerned about the security of my personal information during a telehealth appointment	.66	.01	.72		.12
3. I feel like the quality of care is just as high in a telehealth appointment as it is in a face-to-face appointment	.78	–.15	.76		–.26
4. I would be willing to access health care via telehealth in the future	.82	–.11	.82		–.27
5. Telehealth is useful in exceptional circumstances like COVID-19 but not at other times	–.03	.98	–.04		.97
Eigenvalue	2.32	1.03	2.45		1.01
Total variance explained (%)	46.5	20.5	49.0		20.2

### Predictors of Health Care Avoidance Due to Telehealth

Table 4 shows predictors of health care avoidance due to telehealth at waves 4 and 7.

At wave 4, those who spoke a language other than or in addition to English at home, had a current diagnosis of a medical condition, were younger, exhibited lower telehealth acceptability, and believed that telehealth had limited utility beyond COVID-19 were more likely to avoid health care due to telehealth. At wave 7, younger age, current diagnosis of a medical condition, and low acceptability were similarly related to health care avoidance. Language spoken at home was no longer a significant predictor of health care avoidance at wave 7. However, female participants were more likely to avoid health care due to telehealth at wave 7.

**Table 4:** Predictors of health care avoidance due to telehealth.

	Wave 4			Wave 7	
	Exp(B) (95% CI)	*P* value	Exp(B) (95% CI)		*P* value
Age	*0.98 (0.97-0.99)* ^a^	*.004*	*0.97 (0.96-0.99)*		*<.001*
Live in major city	0.95 (0.58-1.57)	.84	1.29 (0.76-2.20)		.34
Gender (female)	1.25 (0.83-1.90)	.28	*1.65 (1.08-2.53)*		*.02*
Education	1.10 (0.97-1.25)	.13	1.12 (0.98-1.26)		.09
Lives alone	0.76 (0.40-1.44)	.40	0.68 (0.35-1.34)		.27
Language^b^	*1.93 (1.12-3.32)*	*.02*	0.79 (0.42-1.46)		.45
Medical condition	*2.05 (1.32-3.18)*	*.001*	*2.75 (1.75-4.33)*		*<.001*
Tech confidence	0.98 (0.62-1.57)	.94	1.01 (0.63-1.61)		.98
Acceptability	*0.87 (0.81-0.93)*	*<.001*	*0.87 (0.81-0.93)*		*<.001*
Telehealth has limited utility beyond COVID-19	*1.85 (1.20-2.83)*	*.005*	*1.43 (1.15-1.76)*		*.001*

^a^Italicized values are significant at *P*<.05.

^b^Speaks language in addition to/other than English at home.

## Discussion

### Principal Findings

This study found that approximately 30% of participants reported using telehealth during the assessment periods, with the most common telehealth modality being a telephone. Acceptance of telehealth was generally high and was higher among those who used telehealth compared with those who did not. Approximately 18% of participants reported avoiding health care due to telehealth. Across assessment waves, avoidance was associated with younger age, speaking a language other than or in addition to English, having a current medical diagnosis, and lower levels of telehealth acceptability.

There was a major increase in the use of telehealth during the COVID-19 pandemic. Telehealth is an important feature of the health system and has the potential to reduce disparities in access to health care. However, it remains important to understand potential drivers of acceptability, use, and avoidance to address any issues that might inadvertently increase disparities in access. The current data indicate that approximately 30% of participants used telehealth services during the first few months of the pandemic, with more than 80% of these using telephone services. The majority of participants were confident in using telehealth, willing to use telehealth in the future, and had few concerns related to privacy and security. However, there remained a sizable proportion who did have concerns, particularly about whether the quality of telehealth services was as good as face-to-face services. Higher acceptability was consistently found among those who used telehealth during the survey period compared to those who did not. A similar trend has been observed in research targeting mental health care providers [29], suggesting that increased familiarity with telehealth may improve confidence, reduce uncertainty, and address any previously held attitudinal barriers regarding the quality and safety of telehealth.

Avoidance of health care due to telehealth was relatively low at around 18%. This rate is lower than the 25% to 37% rates of delayed or foregone medical care reported in US population studies [30,31], which may partly be explained by access to universal health care in Australia or because our primary focus was on delays specifically related to telehealth. However, at a population level, delays to care and avoidance of health services seen in nearly one in five adults may have considerable long-term impacts on morbidity and potentially mortality. For example, COVID-19–related delays in seeking care for cardiovascular problems, cancer, and mental ill health have been observed in administrative and observational data sets [32-34]. Such delays often magnify inequities (eg, [33]) and have been shown to have negative consequences for treatment outcomes (eg, [35-37]).

Factors associated with avoidance of health care raise further concerns about the likelihood of increasing disparities. Speaking a language other than English, which is a commonly used proxy for minority ethnic groups in Australia, was significantly associated with greater avoidance of telehealth at wave 4. Disparities in engagement with telehealth have similarly been observed among people from different ethnic backgrounds in the United States [26]. This suggests that public health messaging that is both culturally relevant and provided in different languages may be important in reducing health care avoidance. Avoidance among people with health conditions was also consistent with prior literature [30] and may reflect a greater potential need for health care among this group (ie, those without a diagnosis may not have seen a need for health care and were, therefore, less likely to endorse avoidance). Nevertheless, this finding is concerning as those most in need of treatment may have delayed service use during a critical window under the belief that delays would be short-term or temporary. In contrast, the finding that older adults were less likely to avoid treatment due to telehealth is somewhat encouraging, as telehealth research has previously suggested that older adults may be reluctant to engage in telehealth and may face considerable barriers [38]. Gender differences were somewhat unexpected, with women more likely to avoid telehealth than men at wave 7, contrasting with findings that women are typically more likely to engage with health services [39,40]. Our finding suggests that women may be more sensitive to differences between telehealth and face-to-face services, possibly placing greater value on interpersonal interactions in the health care setting [41]. Other recent data from Australia suggested that women delayed accessing telehealth during the pandemic due to not wanting to place a burden on the health system and perceived nonurgency of their health issues [42].

Finally, higher acceptance of telehealth was associated with lower odds of avoiding health care due to telehealth. The scale measuring acceptance included items assessing beliefs about the security and quality of telehealth. This finding suggests that there may be a need to promote the evidence base for the quality of telehealth. Brief patient education about the potential benefits of telehealth may be delivered when setting clinical appointments or through public campaigns to reduce barriers to use. Beneficial messaging may include a focus on ease of use and convenience, along with reassuring patients about the security measures that are incorporated into telehealth services. Half of the participants believed that telehealth had limited utility beyond the pandemic, and only one-third believed it is as high quality as face-to-face services. Holding the belief that the utility of telehealth is limited to the pandemic significantly predicted telehealth avoidance, indicating that perceptions of inferiority still exist and may need to be addressed to reduce disparities in service use.

While the large majority of our sample accessed telehealth via the telephone, emerging technological developments are directed toward video platforms. Current literature suggests that there is a significant cognitive burden associated with video-based platforms, which may increase cognitive load and distract attention away from key messaging due to face view and other features such as pop-up messages [43,44]. Such interference does not occur in phone calls, suggesting that the communication experience may differ between telephone and video-based telehealth in several important ways that impact on communication and cognitive fatigue. These are important avenues for future research.

### Limitations

While there is high uptake of digital technology in the Australian population, our online methodology may have biased our sample toward individuals who are comfortable with technology [45]. Therefore, levels of telehealth avoidance may be higher in the Australian population beyond our sampling pool. Reported levels of avoidance may have also been influenced by our question structure, which omitted a “not applicable” option. It is possible that some participants may have indicated that they were not avoiding telehealth because they had no need for health care at that time. Again, this would have resulted in more conservative estimates of health care avoidance. There was some evidence of variable attrition on several demographic characteristics in the sample, which may also have influenced the findings and the representativeness of the sample used in the analyses.

Further data are needed to explain the potentially counterintuitive finding that people with medical conditions were more likely to avoid telehealth. One possibility is that people with medical conditions reported being more likely to avoid health care because they were more likely to need it. Another possibility is that people with medical conditions prefer face-to-face consultations because they provide other benefits that they do not expect from telehealth. The COVID-19 pandemic may have been perceived as temporary at the time of the survey, and therefore people may have expected they would be able to access their preferred face-to-face services in the near future, contributing to avoidance. Further qualitative research may be needed to explore the contexts in which people avoid or delay treatment and how the mixture of telehealth, digital, and face-to-face services influences avoidance.

Finally, it is possible that the findings of this study may have underestimated the current acceptability of telehealth. This study reports findings from the first lockdown phase of the COVID-19 pandemic in Australia. At this time, over two-thirds of participants had not accessed telehealth services. Exposure to telehealth and associated technologies in other contexts in the months following the initial phases of the pandemic may have improved acceptability further and increased expectations that telehealth will be useful in the future. Similarly, as many services have become more experienced in providing telehealth services, they may be viewed by users as being higher in quality. Further assessment of the impacts and responses to telehealth over time, including during the later stages of the pandemic, may be important to identify emerging gaps in care that could influence equality in service use. In particular, there is a need to investigate how people with complex health needs may respond to telehealth and the changing health service landscape, as other members of the community resume usual health care practices.

### Conclusion

Telehealth use has increased substantially during the COVID-19 pandemic. While most participants in this study were accepting of telehealth services, there remain several key barriers to use. Avoidance of health services due to the introduction of telehealth was seen in approximately 18% of participants in this study, which is less than in US population surveys but still suggests the potential for delays and avoidance of care to have significant impacts on population morbidity and mortality. Some of the factors associated with avoidance of care in this study suggest that disparities in health care use may be increased by the transition to telehealth. To mitigate these potentially negative outcomes, targeted patient education and public health campaigns may be useful for addressing several of the identified barriers, emphasizing the evidence base for telehealth and its ability to deliver care safely and effectively. Structural change may also be needed, ensuring that health policy (particularly rebates for telehealth services), telecommunications infrastructure, and physician education are fit for purpose. There remains considerable potential for telehealth to improve health care access and outcomes, but population trends in use and acceptability must continue to be monitored to ensure that inequity and disadvantage are not inadvertently increased.

## Data Availability

The raw data supporting the conclusions of this paper will be made available by the authors without undue reservation.
